# Agentic AI for interdisciplinary clinical decision support in high-stakes care

**DOI:** 10.1016/j.jhepr.2026.101871

**Published:** 2026-06-10

**Authors:** Fiona R. Kolbinger, Jakob Nikolas Kather

**Affiliations:** 1 Weldon School of Biomedical Engineering, Purdue University, West Lafayette, IN, USA; 2 Department of Visceral, Thoracic and Vascular Surgery, Medical Faculty and University Hospital Dresden, TUD Dresden University of Technology, Dresden, Germany; 3 Else Kroener Fresenius Center for Digital Health, Faculty of Medicine, TUD Dresden University of Technology, Dresden, Germany; 4 Department of Medicine I, Faculty of Medicine, TUD Dresden University of Technology, Dresden, Germany; 5 Medical Oncology, National Center for Tumor Diseases (NCT), University Hospital Heidelberg, Heidelberg, Germany

Hasjim B, Azarfar G, Lee F, Diwan T, Raju S, Gross J, *et al.* A multiagent large language model-based system to simulate the liver transplant selection committee: a retrospective cohort study. The Lancet Digital Health, 2026; 8. 10.1016/j.landig.2025.100966.

The hardest decisions in medicine cannot be described by simple decision trees. Critical care decisions, such as liver transplant allocation, tumor board recommendations, or end-of-life management in intensive care, are examples of complex situations in which good decision-making relies on the synthesis and reconciliation of heterogeneous, sometimes contradictory data, the assessment and weighting of interdisciplinary evidence and expertise, and the prioritization of different treatment options. The first generation of clinical Artificial Intelligence (AI) tools largely addressed simpler, diagnostic problems, such as disease detection and classification, where a single binary prediction based on a single data type maps directly to a clinical action. Examples for such use cases include breast cancer detection on X-ray mammograms or the differential diagnosis of skin lesions (Fig. 1A). Recent clinical trials provide evidence that these tools will be an integral part of future standards of care.[1], [2], [3], [4], [5] However, medical decisions are often so complex that framing them as a one-dimensional prediction task is either impossible or would require an oversimplification of the clinical problem that, ultimately, impedes practical application of the resulting AI tools.^6^^,^^7^

**Fig. 1:**
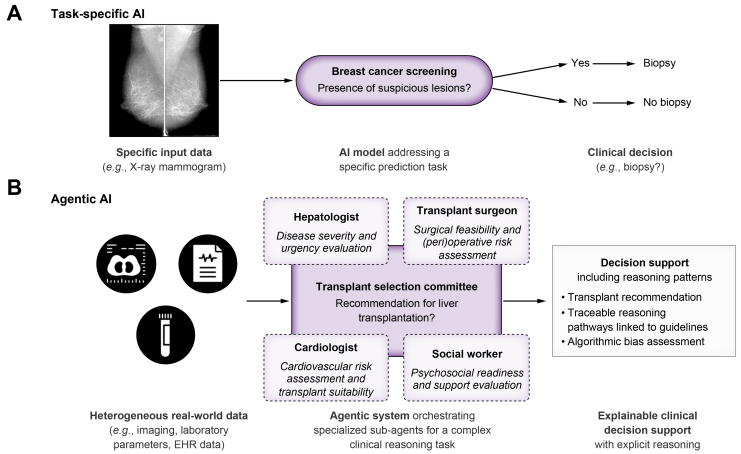
Evolution of medical AI from task-specific algorithms to agentic systems. (A) The first generation of medical AI models addressed clearly defined (*e.g*., diagnostic) tasks with a direct clinical implication. An example is breast cancer screening from X-ray mammograms, where the detection of a suspicious lesion triggers a biopsy. (B) Agentic AI systems can address complex, high-stakes scenarios, such as liver transplant allocation, by coordinating multiple specialized sub-agents. Through simulated multidisciplinary discourse and chain-of-thought reasoning applied to diverse clinical variables, agentic systems output auditable consensus recommendations that facilitate detailed algorithmic fairness and bias analysis.

Technical advances over the past two years have introduced agentic AI systems, a new generation of AI models that is built on large language models (LLMs) and vision-language models.^8^ Agentic AI systems can plan multi-step workflows, call external tools, orchestrate specialized sub-agents, and generate transparent and auditable reasoning traces that provide documentation of why a specific model recommendation was reached. In the context of clinical decision support, this shift opens new AI model capabilities to support complex, debatable, multi-stakeholder decisions (Fig. 1 B).^8^^,^^9^ Here, we highlight a recent study on agentic AI for liver transplant allocation^10^ to illustrate what this repositioning entails in practice and analyze the practical, regulatory, and ethical challenges that currently limit the clinical implementation of agentic AI.

Transplant allocation is a use case that stress-tests clinical AI via several critical boundary conditions: Donor organs are scarce and decisions are multifactorial, value-laden, and heterogeneously implemented internationally and across centers.^11^^,^^12^ In their recent article published in Lancet Digital Health, Hasjim *et al.*^10^ simulate a multidisciplinary liver transplant selection committee using four role-aligned AI agents, a hepatologist, surgeon, cardiologist, and a social worker, who review standardized and AI-summarized vignettes derived from the Scientific Registry of Transplant Recipients, which comprises data on organ transplantation in the United States. The authors then tasked the simulated committee to provide a binary recommendation: either recommend liver transplantation when a 6-month or 1-year survival benefit would be likely, or decline liver transplantation if contraindications exist or if liver transplantation would not offer survival benefits. Across 8,412 clinical cases, the system reported accuracies of 98.2% for identifying absolute contraindications to liver transplantation, 94.9% for 6-month survival benefit, and 92.0% for 1-year survival benefit.

The authors further examined agentic reasoning for each committee role by identifying the most prevalent variables used for respective decisions (by cosine similarity index, a metric indicating alignment between the input variable and the final recommendation) and for whom they decide (using disparate impact, an algorithmic fairness metric). Here, they observed role-specific reasoning priorities (for example, the cardiologist prioritized cardiovascular risk factors) and found that the committee decisions were overall fair. They identified a relatively lower frequency of positive transplant recommendations by the agentic system for female, multiracial, or Hispanic patients, those with end-stage liver disease of biliary etiology, indicating slight disadvantages for these populations. Unexpectedly, patients residing in socioeconomically advantaged areas and patients with grade school education were also less frequently recommended for transplantation.^10^

Two aspects of this study are particularly noteworthy from the perspective of translational hepatology: First, the high predictive accuracy of the agentic committee underscores the potential of AI agents for objective decision-making in the transplant selection process. Second, the authors prioritize algorithmic fairness and the identification of potential biases as critical outcomes to report. Given the risks of hallucinations and the amplification of existing systemic biases through learned priors, bias analysis is of critical importance for agentic AI evaluation. Despite limitations including the retrospective study design, the use of simplified endpoints that do not account for patient-reported outcomes, and reliance on a single modeling and prompting strategy, the study by Hasjim *et al.* provides a timely proof of concept for agentic AI in interdisciplinary transplant committee decision support and highlights its potential to standardize case synthesis and provide a reproducible second read for human committees.

Agentic clinical AI systems are yet to be evaluated in prospective settings. Simulation studies like the work by Hasjim *et al.* demonstrate that agentic architectures and the underlying foundation models are already close to clinical-grade maturity. A recent study evaluating agentic AI in oncology similarly supports the maturity of agentic systems for clinical decision support. This study demonstrates that an agentic AI system for tumor board recommendations integrating LLMs with domain-specific tools, guideline retrieval, and imaging analysis substantially outperforms standalone LLMs in reaching correct clinical conclusions on realistic clinical case vignettes.^13^ However, the integration of such systems into clinical workflows poses relevant practical, regulatory, and ethical challenges.

First, live deployment in real-world clinical settings would demand agentic systems to **handle multimodal electronic health record data that are often unstructured, fragmented, and noisy**. Methods to dynamically extract and synthesize relevant data points from medical records exist,^14^^,^^15^ yet few health systems have currently implemented them in a way that ensures interoperability and data security and would allow for agentic systems to seamlessly interact with patient records. Since 2014, the FHIR (Fast Healthcare Interoperability Resources) have been continuously developed by healthcare informatics stakeholders as a standard and internationally, healthcare systems and industry stakeholders increasingly foster their adoption.^16^^,^^17^

Second, the trajectory of clinical translation of agentic AI will hinge on the **selection of appropriate study designs that comply with regulatory guidelines**.^7^ A complicating factor is that regulatory structures (*e.g*., the European Medical Device Regulation) are often not inherently designed for the iterative agility of agentic architectures, which creates friction for rapid deployment and necessitates innovative environments that allow for controlled evaluation of agents within real-world environments (“living labs”) to identify interoperability hurdles before large-scale rollout.^18^

Third, the autonomous orchestration of complex clinical decisions introduces **ethical and liability challenges**. As demonstrated by the biases detected in the transplant allocation model, agentic systems risk inadvertently amplifying systemic inequities or generating plausible but flawed recommendations. Different implementation scenarios have been designed and analyzed in simpler clinical AI applications, including breast cancer screening,^19^ to address these risks and increase patient and clinician trust. Examples include using the AI-generated recommendation as a second read or triggering expert review of cases triaged as particularly complex or ambiguous. Here, the capability of agentic AI systems to generate verifiable reasoning traces linked directly to source data and clinical guidelines could offer an inherent advantage for explainability and regulatory compliance and help human committees to rapidly audit the logic and confidently assume ultimate accountability for patient outcomes.

The vignette-based designs of recent studies evaluating agentic AI systems for clinical decision support tasks^10^^,^^13^ represent highly controlled proof-of-concept environments, yet clearly demonstrate the potential of agentic AI systems to support complex clinical decisions. Realizing the potential of agentic clinical AI will require close collaboration between clinical teams, software developers, and regulators, to facilitate prospective evaluation in real-world clinical settings. Agentic AI may soon be ready to support clinical decisions. The field of liver disease, where many decisions carry irreversible consequences and existing inequities in clinical care are well documented,^11^^,^^12^ stands among the first and most important testing grounds.

## Authors’ contributions

FRK and JNK conceptualized, wrote, and revised this editorial.

## Financial support

FRK receives support from the 10.13039/100008658German Cancer Research Center (CoBot 2.0), the Central Indiana Corporate Partnership AnalytiXIN Initiative, the Evan and Sue Ann Werling Pancreatic Cancer Research Fund, and the 10.13039/100006975Indiana Clinical and Translational Sciences Institute funded, in part, by the 10.13039/100000002National Institutes of Health, 10.13039/100006108National Center for Advancing Translational Sciences, Clinical and Translational Sciences Award (UM1TR004402). JNK is supported by the 10.13039/501100005972German Cancer Aid DKH (DECADE, 70115166), the German Federal Ministry of Research, Technology and Space BMFTR (PEARL, 01KD2104C; CAMINO, 01EO2101; TRANSFORM LIVER, 031L0312A; TANGERINE, 01KT2302 through ERA-NET Transcan; Come2Data, 16DKZ2044A; DEEP-HCC, 031L0315A; DECIPHER-M, 01KD2420A; NextBIG, 01ZU2402A; PROSURV, 01KD2509C), the 10.13039/501100001659German Research Foundation (DFG, Deutsche Forschungsgemeinschaft) as part of Germany’s Excellence Strategy – EXC 2050/2 – Project ID 390696704 – Cluster of Excellence “Centre for Tactile Internet with Human-in-the-Loop” (CeTI) of Technische Universität Dresden, as well as through DFG-funded collaborative research projects (TRR 412/1, 535081457; SFB 1709/1 2025, 533056198), the 10.13039/100021828German Academic Exchange Service DAAD (SECAI, 57616814), the German Federal Joint Committee G-BA (TransplantKI, 01VSF21048), the 10.13039/501100000780European Union EU’s Horizon Europe research and innovation programme (ODELIA, 101057091; GENIAL, 101096312), the 10.13039/501100000781European Research Council ERC (NADIR, 101114631), the 10.13039/100001006Breast Cancer Research Foundation (BELLADONNA, BCRF-25-225) and the 10.13039/501100000272National Institute for Health and Care Research NIHR (10.13039/501100018955Leeds Biomedical Research Centre, NIHR203331). The content and views expressed are those of the author(s) and not necessarily those of the National Institutes of Health, the NHS, the NIHR or the Department of Health and Social Care. This work was funded by the European Union. Views and opinions expressed are, however, those of the author(s) only and do not necessarily reflect those of the European Union. Neither the European Union nor the granting authority can be held responsible for them.

## Conflicts of interest

FRK declares an ongoing advisory role for the Surgical Data Science Collective, USA. JNK declares ongoing consulting services for AstraZeneca and Bioptimus. Furthermore, he holds shares in StratifAI, Synagen, and Spira Labs, has received institutional research grants from GSK and AstraZeneca, as well as honoraria from AstraZeneca, Bayer, Daiichi Sankyo, Eisai, Janssen, Merck, MSD, BMS, Roche, Pfizer, and Fresenius. JNK is an editor at *JHEP Reports* and was not involved in handling this manuscript.

Please refer to the accompanying ICMJE disclosure forms for further details.
