# Supplementary material for: Agentic AI for interdisciplinary clinical decision support in high-stakes care
Source: JHEP Rep. 2026 Jun 10;8(7):101871. doi: 10.1016/j.jhepr.2026.101871 (PMC13366225; doi:10.1016/j.jhepr.2026.101871)
Supplement: Multimedia component 1 [file mmc1.pdf]

ICMJE DISCLOSURE FORM

Date: May 8, 2026
Your Name: Fiona R. Kolbinger
Manuscript Title: Agentic AI for interdisciplinary clinical decision support in high-stakes care

In the interest of transparency, we ask you to disclose all relationships/activities/interests listed below that are related to the content of your manuscript. "Related" means any relation with for-profit or not-for-profit third parties whose interests may be affected by the content of the manuscript. Disclosure represents a commitment to transparency and does not necessarily indicate a bias. If you are in doubt about whether to list a relationship/activity/interest, it is preferable that you do so.

The following questions apply to the author's relationships/activities/interests as they relate to the current manuscript only.

The author's relationships/activities/interests should be defined broadly. For example, if your manuscript pertains to the epidemiology of hypertension, you should declare all relationships with manufacturers of antihypertensive medication, even if that medication is not mentioned in the manuscript.

In item #1 below, report all support for the work reported in this manuscript without time limit. For all other items, the time frame for disclosure is the past 36 months.

		**Name all entities with whom you have this relationship or indicate none (add rows as needed)**	**Specifications/Comments (e.g., if payments were made to you or to your institution)**
**Time frame: Since the initial planning of the work**			
1	All support for the present manuscript (e.g., funding, provision of study materials, medical writing, article processing charges, etc.) **No time limit for this item.**	Novartis	Institutional grant
**Time frame: past 36 months**			
2	Grants or contracts from any entity (if not indicated in item #1 above).	German Cancer Research Center	Institutional grant (CoBot 2.0)
		Central Indiana Corporate Partnership AnalytiXIN Initiative	Institutional grant
		Evan and Sue Ann Werling Pancreatic Cancer Research Fund	Institutional grant
		Indiana Clinical and Translational Sciences Institute	Institutional grant funded, in part, by the National Institutes of Health, National Center for Advancing

			Translational Sciences, Clinical and Translational Sciences Award (UM1TR004402)
3	Royalties or licenses	None	
4	Consulting fees	Surgical Data Science Collaborative, USA	
5	Payment or honoraria for lectures, presentations, speakers bureaus, manuscript writing or educational events	None	
6	Payment for expert testimony	None	
7	Support for attending meetings and/or travel	American Society for Clinical Oncology, USA	
		Society of Robotic Surgery, USA	
		ORSI Academy, Belgium	
8	Patents planned, issued or pending	None	
9	Participation on a Data Safety Monitoring Board or Advisory Board	None	
10	Leadership or fiduciary role in other board, society, committee or advocacy group, paid or unpaid	None	
11	Stock or stock options	Scopia AI, Canada	
12	Receipt of equipment, materials, drugs, medical writing, gifts or other services	None	
13	Other financial or non-financial interests		

Please place an "X" next to the following statement to indicate your agreement:

__X_ I certify that I have answered every question and have not altered the wording of any of the questions on this form.

ICMJE DISCLOSURE FORM

Date: 5/15/2026

Your Name: Jakob Nikolas Kather

Manuscript Title: Agentic AI for interdisciplinary clinical decision support in high-stakes care

Manuscript Number (if known): Click or tap here to enter text.

In the interest of transparency, we ask you to disclose all relationships/activities/interests listed belo that are related to the content of your manuscript. "Related" means any relation with for-profit or no for-profit third parties whose interests may be affected by the content of the manuscript. Disclosure represents a commitment to transparency and does not necessarily indicate a bias. If you are in doub about whether to list a relationship/activity/interest, it is preferable that you do so.

The author's relationships/activities/interests should be defined broadly. For example, if your manuscript pertains to the epidemiology of hypertension, you should declare all relationships with manufacturers of antihypertensive medication, even if that medication is not mentioned in the manuscript.

In item #1 below, report all support for the work reported in this manuscript without time limit. For all other items, the time frame for disclosure is the past 36 months.

		**Name all entities with whom you have this relationship or indicate none (add rows as needed)**	**Specifications /Comments (e.g., if payments were made to you or to your institution)**
**Time frame: Since the initial planning of the work**			
**1**	All support for the present	☒ **None**	

		Name all entities with whom you have this relationship or indicate none (add rows as needed)	Specifications /Comments (e.g., if payments were made to you or to your institution)
	manuscript (e.g., funding, provision of study materials, medical writing, article processing charges, etc.) **No time limit for this item.**		

Time frame: past 36 months

		Name all entities with whom you have this relationship or indicate none (add rows as needed)	Specifications /Comments (e.g., if payments were made to you or to your institution)
2	Grants or contracts from any entity (if not indicated in item #1 above).	☐ None	
		GSK	
		AstraZeneca	
		German Cancer Aid DKH	
		German Federal Ministry of R[illegible] Technology and Space BMFTR[illegible]	
		German Research Foundation	
		German Academic Exchange S[illegible] DAAD	
		German Federal Joint Commit[illegible]	
		The European Union's Horizo[illegible] research and innovation prog[illegible]	
		European Research Council E[illegible]	
		Breast Cancer Research Foun[illegible]	
		National Institute for Health a[illegible] Research NIHR	

		Name all entities with whom you have this relationship or indicate none (add rows as needed)	Specifications /Comments (e.g., if payments were made to you or to your institution)
3	Royalties or licenses	☒ None	
4	Consulting fees	☐ None	
		AstraZeneca	
		Bioptimus	
		Mindpeak	
		MultiplexDx	
5	Payment or honoraria for lectures, presentations, speakers bureaus, manuscript writing or educational	☐ None	
		AstraZeneca	
		Bayer	
		Daiichi Sankyo	
		Eisai	
		Janssen	
		Merck	
		MSD	
		BMS	
		Roche	
		Pfizer	

		Name all entities with whom you have this relationship or indicate none (add rows as needed)	Specifications /Comments (e.g., if payments were made to you or to your institution)
	events		
6	Payment for expert testimony	☒ None	
7	Support for attending meetings and/or travel	☒ None	
8	Patents planned, issued or pending	☒ None	
9	Participation on a Data	☐ None<br>Astrazeneca<br>DoMoreDiagnostics<br>Owkin	

		Name all entities with whom you have this relationship or indicate none (add rows as needed)	Specifications /Comments (e.g., if payments were made to you or to your institution)
	Safety Monitoring Board or Advisory Board	Panakeia	
10	Leadership or fiduciary role in other board, society, committee or advocacy group, paid or unpaid	☐ None<br>AACR<br>ESMO<br>ESAC<br>EASL<br>DGHO	

		Name all entities with whom you have this relationship or indicate none (add rows as needed)	Specifications /Comments (e.g., if payments were made to you or to your institution)
**11**	Stock or stock options	☐ **None**	
		StratifAI	
		Synagen	
		Spira Labs	
		Tremont AI	
		Saterra AI	
**12**	Receipt of equipment, materials, drugs, medical writing, gifts or other services	☒ **None**	
**13**	Other financial or non-financial	☒ **None**	

	**Name all entities with whom you have this relationship or indicate none (add rows as needed)**	**Specifications /Comments (e.g., if payments were made to you or to your institution)**
inter ests		

Please place an "X" next to the following statement to indicate your agreement:

☒ I certify that I have answered every question and have not altered the wording of any of the questions on this form.
